# What Is the Role of HLA-I on Cancer Derived Extracellular Vesicles? Defining the Challenges in Characterisation and Potential Uses of This Ligandome

**DOI:** 10.3390/ijms222413554

**Published:** 2021-12-17

**Authors:** Caitlin Boyne, Debra Lennox, Olivia Beech, Simon J. Powis, Pankaj Kumar

**Affiliations:** School of Medicine, University of St. Andrews, St. Andrews KY16 9TF, UK; dl89@st-andrews.ac.uk (D.L.); omb4@st-andrews.ac.uk (O.B.); sjp10@st-andrews.ac.uk (S.J.P.); pk20@st-andrews.ac.uk (P.K.)

**Keywords:** HLA-1, extracellular vesicles, immunopeptidome, antigen presentation, mass spectrometry, cancer immunology

## Abstract

The Human Leukocyte Antigen class I (HLA-I) system is an essential part of the immune system that is fundamental to the successful activation of cytotoxic lymphocytes, and an effective subsequent immune attack against both pathogen-infected and cancer cells. The importance of cytotoxic T cell activity and ability to detect foreign cancer-related antigenic peptides has recently been highlighted by the successful application of monoclonal antibody-based checkpoint inhibitors as novel immune therapies. Thus, there is an increased interest in fully characterising the repertoire of peptides that are being presented to cytotoxic CD8+ T cells by cancer cells. However, HLA-I is also known to be present on the surface of extracellular vesicles, which are released by most if not all cancer cells. Whilst the peptide ligandome presented by cell surface HLA class I molecules on cancer cells has been studied extensively, the ligandome of extracellular vesicles remains relatively poorly defined. Here, we will describe the current understanding of the HLA-I peptide ligandome and its role on cancer-derived extracellular vesicles, and evaluate the aspects of the system that have the potential to advance immune-based therapeutic approaches for the effective treatment of cancer.

## 1. Extracellular Vesicles

**Figure 1:**
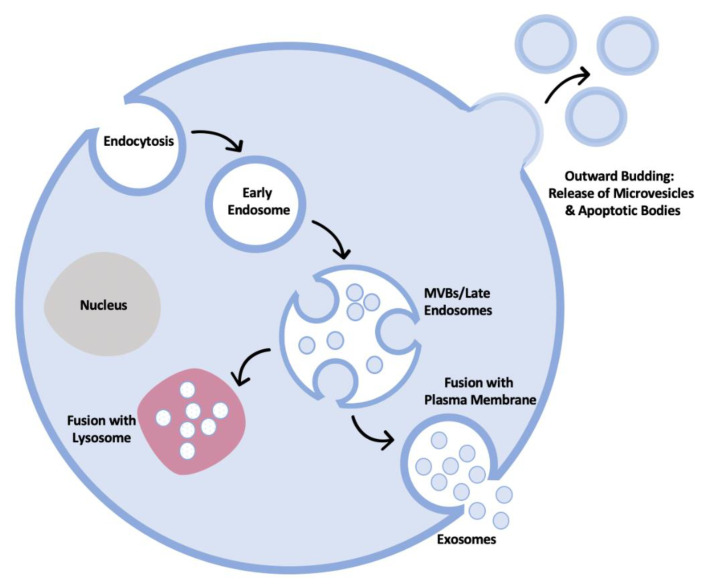
Representation of the several routes known for extracellular vesicle (EV) biogenesis. EVs can be released in the form of microvesicles generated from the outward budding of the plasma membrane, or as exosomes through the fusion of multivesicular bodies (MVB) from within the endosomal/lysosomal pathway with the plasma membrane. MVBs can alternatively degrade their cargo by fusing with lysosomes. Similar to that of microvesicles, apoptotic bodies are also released by outward budding in cells that are undergoing apoptosis.

Extracellular vesicles (EVs) have recently become of notable interest due to their role in the mediation of intercellular communication and, in particular, their role in cancer [1]. It has been demonstrated that EVs play a role in a large majority of cancer progression steps. However, there is still a lack in understanding as to the extent of their function in these processes, and which cancer-specific pathways impact upon EV biogenesis and function [2]. The biogenesis of EVs is variable and results in different categories of vesicles, including microvesicles, apoptotic bodies and exosomes [3]. The size range of EVs is wide, typically encompassing between 30–1000 nm, but most work has been carried out on populations in the 50–200 nm range. Microvesicles are formed by the direct outward budding of the plasma membrane and are slightly larger EVs with a size range of 150–1000 nm, whilst apoptotic bodies are formed during the process of fragmentation that occurs to cells during programmed cell death and results in the largest class of EVs at 1000–5000 nm [4]. Exosomes are the smallest of the populations at around 40–150 nm, formed via the inward budding of endosomal membranes, resulting in the formation of multivesicular bodies (MVBs). These MVBs contain intralumenal vesicles, which are released upon fusion with the plasma membrane as exosomes (Figure 1) [5]. The content of EVs varies substantially and is largely dependent on their cell of origin, however, they are capable of carrying various proteins, lipids, DNA and RNAs [6].

Under homeostatic conditions, EVs aid the process of intercellular communication through the transference of their contents between cells, resulting in behavioural changes of the recipient cells [7]. Additionally, EVs have been shown to be involved in a vast number of pathological and physiological processes and have also been implicated in the development of a number of neurological and cardiovascular diseases [8]. However, in the context of this current review it is significant that they are involved in almost every step of progression required for the development of carcinomas from the initial stage of the primary tumour through to metastasis. Numerous studies have now confirmed that there is a distinct difference between normal cell exosomes and cancer cell exosomes, both in terms of their content and the rate at which they are released [9,10,11].

The content of tumour-derived EVs is diverse, can vary considerably and is largely dependent on the cell of origin, in a similar manner to normal cell EVs. However, the contents are highly bioactive and are able to elicit a considerable change in the activity of the recipient cells. Some of the molecules carried by cancer-derived EVs include oncoproteins and oncopeptides, as well as various RNA and DNA species that have the capacity to instigate substantial changes to the tumour microenvironment. These changes often aid the progression of tumour and metastasis development as EVs have been known to feature a role in aspects of invasion and the maintenance of angiogenesis [12,13,14]. EVs have been described to release factors involved in the epithelial to mesenchymal transition process via stimulation of the epithelial cells, resulting in increased angiogenesis, extracellular matrix remodelling and the loss of adhesion of tumour cells. This change in the microenvironment encourages the release of tumour cells into circulation whereby they have the ability to travel to alternative locations within the host and form metastases [14]. Additionally, EVs have the ability to contribute towards the development of an aggressive disease phenotype and the development of chemo and radio-therapy resistance [15]. Extensive evidence has shown that one of the principal actions of cancer-derived EVs is to inhibit the anti-tumour immune response. More specifically, studies have shown that cancer-derived EVs have the ability to interrupt the recruitment, activation and functionality of a number of key immune cells, including macrophages, regulatory T cells (Tregs), MDSCs, dendritic cells and cytotoxic CD8+ T cells [2,16,17]. Evidently, EVs play a number of important roles that make them excellent targets for novel immunotherapeutic techniques and for biomarker mining [18].

## 2. Role of HLA-I in Cells

**Figure 2:**
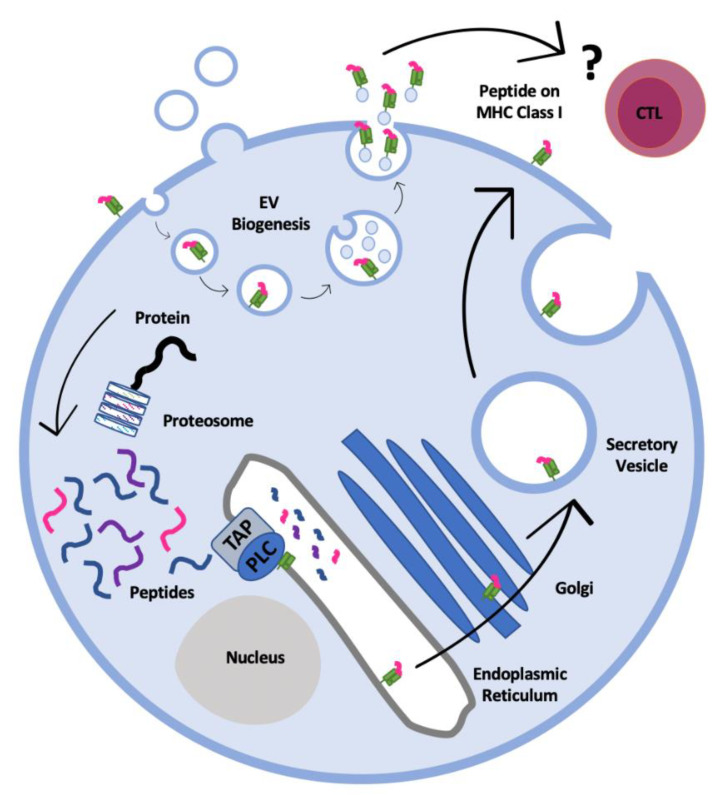
An outline of the human leukocyte antigen (HLA)/major histocompatibility complex (MHC) class I presentation pathway. The majority of the degradation of proteins destined for antigen presentation by HLA-I occurs in the cytosol by the proteasome. These peptides are then translocated into the lumen of the endoplasmic reticulum (ER), by the transporter complex associated with antigen processing (TAP), where further trimming may occur. Peptides are then loaded onto HLA-I molecules with the help of chaperones and the resulting stable complexes are transported from the endoplasmic reticulum to the surface of the cell. The incorporation of HLA-I molecules into exosomes generated through the MVB pathway is thought to occur by internalisation of HLA-I molecules from the cell surface.

Human leukocyte antigen I (HLA-I) and II (HLA-II) refers to the major histocompatibility complex (MHC) class I and class II proteins found in humans. Whilst HLA-II molecules are restricted in expression and are typically only found on B cells and antigen presenting cells and are responsible for the presentation of antigens to CD4+ T cells, HLA-I molecules can be detected on the surface of almost all nucleated cells and are essential for the activation of a CD8+ T cell response [19]. The critical role played by HLA-I antigen presentation has been demonstrated where the possession of HLA-I heterogeneity, and also carrying tumours with a high mutational burden, leads to significantly increased survival times after checkpoint blockade therapy [20]. Tumour antigens are normally classed as either tumour-specific antigens (TSA) which are expressed exclusively by tumour cells, or as tumour-associated antigens (TAA), which can be expressed by normal cells but are known to frequently be overexpressed in tumour tissue [21]. Eliciting a T cell response requires the presentation of peptides which are generated primarily by proteasomal degradation in the cytosol. Subsequent transport by transporter associated with antigen processing (TAP) into the lumen of the endoplasmic reticulum and further trimming by proteolytic enzymes such as endoplasmic reticulum aminopeptidase (ERAP) allows selected binding by HLA-I molecules. These peptides can then be presented to antigen specific cytotoxic CD8+ T cells (Figure 2), and in the context of cancer, lead to significant eradication of tumour cells. The increased development and use of monoclonal antibody checkpoint inhibitor therapies (directed against molecules such as PDL-1 and CTLA-4) have highlighted the benefits that can result when the ability of CD8+ T cells to recognise cancer-related antigenic peptides on the surface of cells is enhanced.

## 3. Immunotherapeutic Potential of EVs

The presence of HLA-I molecules on the surface of EVs and the knowledge of their ability to present antigenic peptides to T cells has been well established [22,23,24]. Whilst the peptide ligandome presented by cell surface HLA-I molecules on cancer cells has been extensively investigated, the peptide ligandome on EVs is yet to be wholly defined. The fact that HLA-I molecules are frequently present on EVs released by cancer cells raises the possibility that they may be used as an additional or alternative source of material to characterise and mine the HLA-I peptide ligandome relevant to anti-cancer responses. The presence of EVs in the bloodstream [25] further raises their potential to help characterise HLA-I relevant TAA or TSA of cancers that are difficult or impossible to biopsy to generate solid tissue generated ligandomes. A key question that then arises is whether or not the HLA-I peptide ligandome repertoire of EVs is a full representative duplicate of that of the cell surface. In our first study of EV HLA-I ligandomes, we utilised the Epstein-Barr virus-transformed B cell line Jesthom to address this question. HLA-I binding peptides were identified and analysed by carrying out small-scale isolations of MHC class I molecules on EVs and comparing the resulting peptide repertoire to that of the cell surface [26]. A total of 516 peptides were eluted from both the cell surface and EVs that had predicted binding affinities to HLA-A*02:01 or HLA-B*27:05. Between the two groups of peptides isolated, there were no significant differences in either the resulting peptide-anchor motifs or the range of predicted serotype-binding affinities. This study provided the first set of data to confirm that EVs could effectively provide valuable information on the peptides present on HLA-I and could be assessed as essentially duplicates of the cell surface HLA-I ligandome. From this initial study we hypothesized that EVs could be of substantial use in cancer studies with important antigenic peptides, including TAA and TSA, likely to be released on EVs and therefore identifiable through mass spectrometry of their HLA-I ligandomes.

To directly address the above question, we have recently completed a study using multiple cell lines representing breast cancer, melanoma and myeloma (Kumar et al., unpublished). Over 9000 peptides were characterised from the cell surface and EV HLA-I ligandomes combined, and we were able to detect over 100 peptides in the EV ligandome which were derived from tumour-associated proteins. In fact, over 5% of the peptides detected in the EV HLA-I ligandome were derived from tumour antigens, indicating their high relative abundance. Additionally, we identified a number of mutated peptides that could be potential TSA. This new study shows direct evidence of the presence of clinically relevant tumour-associated antigenic peptides in the HLA class I ligandome present on EVs.

**Figure 3:**
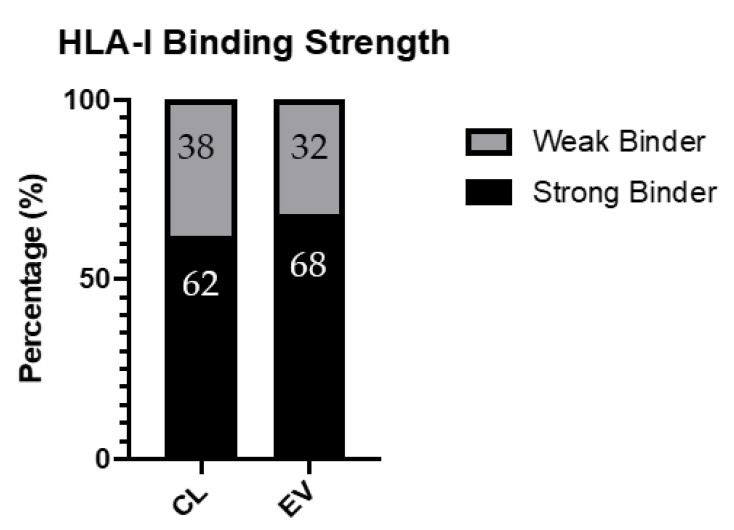
Predicted binding affinities for cell surface (CL) and extracellular vesicle (EV) HLA-I peptides isolated from cancer cells lines. Based on predicted binding affinity by NetMHCpan 4.0 [28], HLA-I peptides were classified as strong binder (binding affinity less than 100 nM) or weak binders (binding affinity over 100 nM and up to 1000 nM) and the percentage of strong and weak binding peptides were plotted for both the cell and EV HLA-I ligandomes. The numbers in the columns indicate the percentages of each subset. The EV HLA-I peptide pool contained more peptides with predicted strong binding affinities compared to the cell surface HLA-I peptide pool.

Our new extended dataset of peptides also allowed us to confirm an observation on HLA-I ligand affinity made in our previous study on Jesthom cells. We classified peptide ligands from cells and EVs as strong binders (predicted HLA-I binding affinity less than 100 nM) or weak binders (predicted HLA-I binding affinity between 100 nM and 1000 nM), respectively. A higher proportion of strong binding peptides were detected in the EV ligandome compared to the cell ligandome (Figure 3). At present, we do not fully understand why this should be the case, but one intriguing possibility is that the exosome portion of the EVs has passaged through the MVB compartment within cells and may have been subjected to an HLA-I peptide ‘editing’ environment that has not been fully characterised. As a consequence, some of the lower affinity peptides may have been lost. This also implies the possibility of MVB-derived unique peptides also being loaded onto HLA-I in an exchange process within the MVB. Both of these possibilities require further study.

The detection of TAA and potential TSA amongst the EV HLA-I ligandome raises the question of whether such antigenic peptides could activate specific CD8+ T cells. If so, cancer cell-derived EVs could be acting as decoys, causing ′off-target′ activation of T cells away from the main tumour mass, limiting the anti-cancer response. There are relatively few studies that describe the ability of HLA-I molecules on EVs to directly promote the activity of cytotoxic CD8+ T cells, however, one study presented an increase in the interferon gamma secretion from purified CD8+ T cells when incubated with EVs loaded with a selection of common antigenic viral peptides [27].

## 4. Challenges of Mapping the HLA-I Ligandome of EVs

Defining the HLA-I ligandome of EVs usually involves the large-scale isolation of HLA-I molecules and mass spectrometric analysis of the resulting peptide pools. This is currently a major challenge as there are a number of variations in EV isolation protocols. In addition, defining the HLA-I ligandome of cancer-derived EVs holds additional challenges as the ratio of cancer-derived EVs to normal EVs will likely vary substantially and the number of peptides that result will therefore be low in number. The characterisation of biomarkers for cancer-specific EV would assist in the isolation of EV cancer subsets, for example by monoclonal antibody affinity purification, but again detection limits by current mass spectrometric methods and limits to the amount of patient material that can be accessed pose significant challenges that need to be addressed. Typically, HLA class I peptide isolation from both cell and EV lysates is carried out using immunoprecipitation, whereby the lysates are subjected to immunoaffinity chromatography, typically using the pan-HLA class I antibody W6/32 [29,30,31]. Preferably, the antibody is crosslinked to agarose resins through covalent coupling to Protein A or Protein G. The HLA-I molecules are then eluted using an acid buffer, for example, TFA or acetic acid [32,33]. The released peptides are then enriched away from the HLA-I heavy chain and B2m proteins by C18-matrix or other HPLC fractionation and elution with a moderate concentration of acetonitrile that favours elution of the peptides over HLA-I components. Alternatively, ultrafiltration that relies on the application of a molecular weight filter can be applied to obtain a sub-3000 Dalton fraction [29,31]. Peptide sequences are identified by liquid chromatography-coupled tandem mass spectrometry (LC-MS/MS), whereby the sequencing is typically achieved through the use of collision induced dissociation (CID) or by higher energy collisional dissociation (HCD) [30]. The resulting fragmented peptide ions can then be searched using various automated search databases, and additionally can be applied to de novo analysis to identify the peptide sequences. These peptide sequences can then be searched against a number of databases to source their matched proteins and potential TAA and TSA can be identified [30,32]. In recent years it has also been recognised that a substantial proportion of HLA-I ligands are in fact generated from non-canonical sources such as non-coding regions and from peptide-splicing events occurring within the proteasome [34,35,36]. This emerging picture of neoantigens will require the adoption and use of novel databases to ensure the full range of the HLA-I ligandome is mapped.

Whilst the field of large-scale immunopeptidomics is rapidly evolving, there is substantial room and broad scope for standardising protocols wherever possible amongst the many laboratories generating HLA-I peptidome data. If the ideals of the Human Immunopeptidome Project are to be realised [37], where immunopeptidome data has been proposed to be useful in the same manner as proteome and genome wide association studies (GWAS), a higher degree of standardisation will be a requisite. Typical current issues with mass spectrometric instrumentation, over and above sample preparation, can include sensitivity, resolving power and acquisition speeds. Sensitivity is a particular issue for smaller samples, which would be the typical situation for many clinical samples, as only the most abundant and easily detected peptides are identified [38]. There are also a number of restrictions beyond the isolation of peptides related to the analysis and the ability to match peptides to proteins effectively and identify antigens of interest, largely due to a lack of complete databases and efficient algorithms for searching the raw LC-MS/MS data. In addition, the methodology of data independent acquisition (DIA) has also been utilised to supplement the more traditional data dependent acquisition (DDA) modes and may prove to be of significant value. Thus, it is likely that the efficiency and accuracy of future immunopeptidomic studies will rely heavily on the establishment of simple and refined protocols aimed at reducing the loss of peptides, further development of DIA protocols to achieve more sensitivity and reproducibility, and on the continuous improvement of instrument sensitivity. Ultimately, mass spectrometric characterisation of peptides may be supplemented with novel methods of single peptide sequencing that could, in combination, fully map the entire immunopeptidome of any given sample [39,40,41].

## 5. Potential Manipulation of EVs

Tumour-derived exosomes could be ideal candidates for an efficient tumour antigen delivery system that would result in the activation of cytotoxic lymphocytes and enhance anti-tumour protection. EVs are an attractive option as they would be readily available and easily accessible through a number of standard biological samples such as blood, urine and cerebrospinal fluid. The peptidomic information that could be retrieved from EVs could provide valuable insight into the immune-modulatory actions being exercised by tumour cells. In theory, similar repertoires of peptides and associated TAA/TSA should be observed on the MHC class I molecules of EVs derived from the same cancer cells. As a result, patterns of peptide expression could lead to diagnostic and prognostic biomarkers and could additionally provide targets for a form of immune therapy or cancer vaccine [18,42,43,44]. The search for appropriate cancer vaccines is a continuously developing field [45]. An effective cancer vaccine requires the ability to elicit an immune response characterised by tumour antigen-specific cytotoxic T-lymphocytes that are capable of recognising and acting upon residual or metastatic tumour cells. In effect, the ideal cancer vaccine would enhance the presentation of antigens by antigen presenting cells, resulting in the promotion of an effective anti-tumour immune response. Current challenges that are associated with the search for an effective cancer vaccine include a lack of available tumour antigens that are both relevant and known to invoke an immune response, as well as a lack of effective delivery or targeting system. Defining the HLA-I ligandome of EVs derived from cancer cells could provide the specific tumour antigens that would be ideal targets for a cancer vaccine.

Thus far, research has focused on the loading of dendritic cell-derived EVs to deliver tumour antigens as they are assumed to be able to effectively maintain their homeostatic functions of presenting TAA and promoting a TAA-specific immune response. A number of clinical trials have been conducted over the years using TAA-loaded dendritic cell-derived EVs, with varying results [46,47]. One early phase I clinical study loaded melanoma-associated tumour antigens (MAGEs) onto dendritic cell-derived EVs and applied them therapeutically to advanced melanoma patients with MAGE3+ disease characterisation. Of the 15 patients involved in the study, one elicited a response, one elicited a minor response and two elicited disease stabilisations, although no MAGE-specific T cell responses were recorded [46]. A different group conducted a similar study on patients diagnosed with advanced MAGE+ non-small cell lung cancer and found that over a third of the participants effectively developed a MAGE-specific immune response as a result of the therapeutic application of MAGE loaded dendritic cell-derived EVs [47]. Another Phase I clinical trial using the vaccination of dendritic cell-derived exosomes in patients diagnosed with advanced melanoma resulted in the successful promotion of activation and proliferation of natural killer cells [48]. Additionally, in 2016, Besse and colleagues also confirmed that dendritic cell-derived exosomes improved the activity of natural killer cells through the results of a Phase II clinical trial on patients diagnosed with non-small cell lung cancer [49]. The adaptation of cancer vaccines to target patient TAA or TSA would be a form of personalised immune therapeutics involving the formation and injection of synthetic versions of the mutated peptides loaded on to extracellular vesicles. Whilst the therapeutic use of EVs as a targeted delivery system has only been implemented in early trials, the results collected thus far have established that the use of EVs as vectors is a safe and viable option, with substantial potential in manipulating and enhancing the immune response in a variety of cancers.

## 6. Conclusions

The essential role of HLA-I in the presentation of peptides to CD8+ T cells provides a promising target for immune therapies, especially in the field of cancer. Additionally, the repertoire of peptides expressed on the HLA-I ligandome of cancer-derived EVs could provide a rich source of information that could be utilised to develop innovative and novel approaches to both personalised immune therapy and cancer diagnostics. Additional studies into EV HLA-I ligandomes, investigating the effects of variables such as the stage of cancer, location of primary and metastatic lesions, and the impact of treatment regimens on the resulting immunopeptidome of EVs, will further our understanding of how the peptide landscape changes throughout the course of the disease and could potentially define the most favourable methods or timepoints to intervene. Recent literature also demonstrates the need to establish standardized approaches to identifying HLA-I peptides to reduce variabilities between peptidomic studies in both sample preparations and the subsequent mass spectrometric analysis of immunopeptidomes.
